# Supplementary material for: Characterization of Vibrio cholerae isolates from freshwater sources in northwest Ohio
Source: PLoS One. 2020 Sep 3;15(9):e0238438. doi: 10.1371/journal.pone.0238438 (PMC7470319; doi:10.1371/journal.pone.0238438)
Supplement: S1 Table — (PDF) [file pone.0238438.s001.pdf]

Collection site	Geographical coordinates
**IV**	41° 36’ 4” N<br>83° 34’ 8” W
**UP**	41° 33’ 37” N<br>83° 38’ 35” W
**S**	41° 39’ 1” N<br>83° 31’57” W
**D**	41° 33’ 41” N<br>83° 37’ 52” W
**B**	41° 41’ 28” N<br>83° 25’ 51” W
**MB**	41° 41’ 8” N<br>83° 22’ 46” W
