# Supplementary material for: Characterization of Vibrio cholerae isolates from freshwater sources in northwest Ohio
Source: PLoS One. 2020 Sep 3;15(9):e0238438. doi: 10.1371/journal.pone.0238438 (PMC7470319; doi:10.1371/journal.pone.0238438)
Supplement: S2 Table — (PDF) [file pone.0238438.s002.pdf]

**Primer**	**Sequence (5'-3')**
ompW F	CACCAAGAAGGTGACTTTATTGTG
ompW R	GAACTTATAACCACCCGCG
ompU F	ACGCTGACGGAATCAACCAAAG
ompU R	GCGGAAGTTTGGCTTGAAGTAG
ctxA F	CGGGCAGATTCTAGACCTCCTG
ctxA R	CGATGATCTTGGAGCATTCCCAC
ctxB F	ATGCACATGGAACACCTCAAAATATTACTG
ctxB R	TCCTCAGGGTATCCTTCATCCTTTCAATC
rtxA F	CTGAATATGAGTGGGTGACTTACG
rtxA R	GTGTATTGTTCGATATCCGCTACG
rxtC F	TGCAAATCTCACATTAGCGCA
rtxC R	CCACTGCACCTTTCGGATACA
hlyA F (classical)	GGCAAACAGCGAAACAAATACC
hlyA F (El Tor)	GAGCCGGCATTCATCTGAAT
hlyA R	CTCAGCGGGCTAATACGGTTTA
tcpA F	CACGATAAGAAAACCGGTCAAGAG
tcpA R (classical)	TTACCAAATGCAACGCCGAATG
tcpA R (El Tor/O139)	CGAAAGCACCTTCTTTCACACGTTG
tcpI F	TAGCCTTAGTTCTCAGCAGGCA
tcpI R	GGCAATAGTGTCGAGCTCGTTA
ace F	TAAGGATGTGCTTATGATGGACACCC
ace R	CGTGATGAATAAAGATACTCATAGG
st F	GAGAAACCTATTCATTGC
st R	GCAAGCTGGATTGCAAC
zot F	TCGCTTAACGATGGCGCGTTTT
zot R	AACCCCGTTTCACTTCTACCCA
